# Six key topics informal carers of patients with breathlessness in advanced disease want to learn about and why: MRC phase I study to inform an educational intervention

**DOI:** 10.1371/journal.pone.0177081

**Published:** 2017-05-05

**Authors:** Morag Farquhar, Clarissa Penfold, John Benson, Roberta Lovick, Ravi Mahadeva, Sophie Howson, Julie Burkin, Sara Booth, David Gilligan, Christopher Todd, Gail Ewing

**Affiliations:** 1 School of Health Sciences, University of East Anglia, Norwich, United Kingdom; 2 Department of Public Health and Primary Care, University of Cambridge, Cambridge, United Kingdom; 3 Lay carer representative, Norwich, United Kingdom; 4 Department of Respiratory Medicine, Cambridge University Hospitals NHS Trust, Cambridge, United Kingdom; 5 SK Nurses, St Ives, Cambridgeshire, United Kingdom; 6 Palliative Care Team, Cambridge University Hospitals NHS Trust, Cambridge, United Kingdom; 7 Department of Oncology, University of Cambridge, Cambridge, United Kingdom; 8 School of Nursing, Midwifery and Social Work, The University of Manchester, Manchester, United Kingdom; 9 Centre for Family Research, University of Cambridge, Cambridge, United Kingdom; Public Library of Science, FRANCE

## Abstract

**Introduction:**

Breathlessness is a common symptom of advanced disease placing a huge burden on patients, health systems and informal carers (families and friends providing daily help and support). It causes distress and isolation. Carers provide complex personal, practical and emotional support yet often feel ill-prepared to care. They lack knowledge and confidence in their caring role. The need to educate carers and families about breathlessness is established, yet we lack robustly developed carer-targeted educational interventions to meet their needs.

**Methods:**

We conducted a qualitative interview study with twenty five purposively-sampled patient-carer dyads living with breathlessness in advanced disease (half living with advanced cancer and half with advanced chronic obstructive pulmonary disease (COPD). We sought to identify carers’ educational needs (including what they wanted to learn about) and explore differences by diagnostic group in order to inform an educational intervention for carers of patients with breathlessness in advanced disease.

**Results:**

There was a strong desire among carers for an educational intervention on breathlessness. Six key topics emerged as salient for them: 1) understanding breathlessness, 2) managing anxiety, panic and breathlessness, 3) managing infections, 4) keeping active, 5) living positively and 6) knowing what to expect in the future. A cross-cutting theme was relationship management: there were tensions within dyads resulting from mismatched expectations related to most topics. Carers felt that knowledge-gains would not only help them to support the patient better, but also help them to manage their own frustrations, anxieties, and quality of life. Different drivers for education need were identified by diagnostic group, possibly related to differences in caring role duration and resulting impacts.

**Conclusion:**

Meeting the educational needs of carers requires robustly developed and evaluated interventions. This study provides the evidence-base for the content of an educational intervention for carers of patients with breathlessness in advanced disease.

## Introduction

Breathlessness is a common devastating symptom of advanced cancer and non-malignant disease [1]. Its incidence in cancer is second only to that of pain [2], occurring in 49% of the general population with all cancers [3] (a figure set to rise in relation to mesothelioma [4]) and 90% of those with lung cancer [5]. It is nearly universal in chronic obstructive pulmonary disease (COPD) by the time of death. COPD is the fourth leading cause of death worldwide [6], predicted to rise to third by 2020 [7]; in the UK alone it affects over 3 million people [8]. Non-pharmacological palliative interventions are effective management strategies for breathlessness but are unevenly provided outside specialist palliative care [9].

Breathlessness places a huge burden on patients, health systems, and on informal carers (family members and friends providing daily help and support) causing distress and isolation [10]. There is limited public understanding of it [11]. Data on the number of carers supporting patients with breathlessness is scarce but presence of a carer has been reported for 60% of patients with advanced cancer-related breathlessness [12] and 66%-77% of patients with advanced COPD [13–15] (data from UK [12–14] and Norway [15]).

Carers of patients with breathlessness due to advanced disease provide complex personal care (e.g. washing, dressing, managing symptoms by administering medication or oxygen) as well as practical and emotional support; their roles are multiple [13], often requiring overnight vigilance [10,16]. They play a crucial role in providing supportive care [17] but also reduce formal statutory social care costs [18–20] and contribute to the social care economy [21]. There are substantial effects on carers’ own health [22], but the reality of caring often means putting their own health second [16].

Carers’ experiences and needs in relation to breathlessness include anxiety and emotional distress, isolation, personal restrictions, lack of knowledge and strategies, helplessness and powerlessness, and lack of support or assistance [10,12,13,14,22,23]. Caring for someone with breathlessness is a difficult role full of uncertainty and the need to manage negative reactions from others, person loss (coping with personality changes), and (in the context of COPD) acute exacerbations [22]. Carers live in an “imploded world” [22 –p156] where they are challenged by breathlessness and lack strategies to relieve the symptom [22]. It is expected that health care professionals will receive training on managing patient symptoms, but many carers receive no such guidance and feel ill-prepared to care [24,25]. This is a source of anxiety which can impact on carer confidence [26] and ability to ask for help, leading to crises: fear of breathlessness drives demand for emergency health care, some of which is unwarranted [27], and may not necessarily address the causes of presentation for help.

A recent study found that more than half of carers of patients with advanced COPD wanted more support with knowing what to expect in the future, and more than a third wanted more support with understanding the illness, knowing who to contact when concerned, having time for themselves in the day and dealing with their feelings and worries [13]. More than half felt unprepared for finding out about and setting up services, and more than a third felt unprepared for getting help and information from the health system, and for responding to and handling breathlessness [16]. Carers experience feelings of uncertainty about the possible trajectory of deterioration in patient health, what the future holds and fear associated with acute exacerbations [22]: they want support to better manage symptoms such as breathlessness [10,28].

We lack robustly developed and tested evidence-based interventions for carers [29]. There is insufficient focus on assisting carers to acquire information and practical skills in cancer [25], at the end of life [30,31], in chronic disease [32] and, more specifically, in COPD [33–37] and in relation to breathlessness in advanced disease [38]. The American Thoracic Society has noted the importance of education for carers and families, as well as for the patient, in managing breathlessness [39]. A recent UK online survey revealed an appetite amongst clinicians for an educational intervention for carers of patients with breathlessness in advanced disease [40]. Educating carers about breathlessness and evidence-based non-pharmacological interventions for breathlessness [9] may meet both patient and carer needs by both upskilling carers and increasing their confidence such that their ability to identify and respond to patient need is enhanced, potentially increasing their satisfaction with the caring role.

The Learning about Breathlessness Study programme is taking an approach based on the Medical Research Council (MRC) framework for complex interventions [41] and the principles of educational planning [42] to develop and evaluate an educational intervention for carers of patients with breathlessness in advanced disease. The study reported here is a component of the MRC Phase I of the programme: its aim is to identify the educational needs of carers of patients with breathlessness due to advanced disease (i.e. to establish what these carers want to learn about) in order to provide an evidence base for the intervention’s content.

## Methods

### Study design

A qualitative in-depth interview study with patient-carer dyads living with breathlessness in advanced disease.

### Ethics

The study received ethics approval from the National Research Ethics Service (NRES) Committee East of England—Cambridge Central (Reference number 14/EE/0009). Written informed consent was obtained from all participants. The study protocol enabled the research team to refer patients or carers to the palliative care Cambridge Breathlessness Intervention Service (BIS) and/or an emotional support contact if required (JuB, a highly experienced occupational therapist with BIS).

### Recruitment

Given the known disease-specific differences in trajectories of patients with breathlessness [43], their experience of breathlessness [10,44–46] and trajectories of carers [47,48], we anticipated that the educational needs and preferences of informal carers were likely to differ by patient disease group. Patients with cancer and intractable breathlessness usually have a relatively short time to live, whereas in COPD the experience of living with breathlessness is prolonged [10,44–46]. Thus the study sought to recruit patient-carer dyads from the two differing disease groups (cancer and COPD) to enable identification of any disease-specific requirements for an intervention’s content and/or mode of delivery. A sample of up to 30 patient-carer dyads was sought, with data collection stopping once theoretical saturation was reached. This maximum sample size was in-line with other qualitative studies of this nature, enabling in-depth exploration of the experiences, needs and preferences sought, as well as analysis by disease group.

Recruitment to palliative care studies [49] and recruitment of carers [50–52] can be difficult, thus we sought patient-carer dyads through multiple care settings: primary care (10 sites), secondary oncology care and secondary respiratory care (six secondary care sites). Dyads were recruited via the patient, and patients were identified by their direct care team (primary or secondary care practitioners providing care to patients who had agreed to facilitate study recruitment). Patients were eligible for inclusion if they had a diagnosis of cancer/COPD and were troubled by breathlessness in spite of optimisation of underlying illness. Patients were excluded if they were aged under 18 years or unable to give informed consent. These pragmatic study entry criteria were adopted to reflect the real world of ultimate intervention adoption where referral to, or uptake of, the intervention is more likely to be related to need for learning about breathlessness than to factors such as the severity of patient breathlessness or the number of hours the carer spends caring.

Patients were sent a recruitment pack by their direct care team (primary or secondary care), consisting of a letter of invitation, participant information sheet, reply slip and freepost envelope for reply directly to the study team. The study team then telephoned responding patients to answer any questions they had and to establish if the patient had an informal carer. Carers were eligible for inclusion if they were identified by the patient as a family member or friend providing them with help or support; they were excluded if they were aged under 18 years or unable to give informed consent.

### Data collection

Interviews with consented patients and carers were conducted in the dyad’s place of choice (usually the patients’ home), by CP/MF. Patients and carers were interviewed separately where possible and audio-recorded, with permission. Interviews lasted about 45 minutes for patients and about an hour for carers. A topic guide was informed by the existing literature, pilot work with patients and carers, and the study’s Monitoring, Advisory & Working Group (MAWG). The guide was reviewed after initial interviews and minor adjustments made.

Patient interviews began by asking for a brief account of their breathlessness and underlying condition to provide context and understand its impact. The interview then explored how patients managed their breathlessness, how they had learnt to manage it and whether they had sought information from sources other than via health care professionals (e.g. via the internet or support groups). Patients were then asked to give a brief account of the carer’s role, how the carer helped them when they were breathless, how the carer had learnt this and whether the carer had sought information from other sources; whether or not patients wanted help from the carer was then explored. Patients were asked whether they wanted to learn (more) about breathlessness, what they thought the carer wanted to learn about, what they wanted the carer to know more about, and whether they wanted to learn with the carer.

Carer interviews began by asking for a brief account of the patient’s breathlessness, the carer’s role, how they helped the patient when they were breathless, how the carer had learnt this and whether the carer had sought information from other sources. Carers were then asked about their own support needs in relation to the patient’s breathlessness, and how prepared they felt to care for the patient. The carer’s experience of responding to and handling the patient’s breathlessness was explored: what they found difficult, whether anything worried or frightened them, what they felt confident about, and what they felt unsure about. Finally, carers were asked about their preferences for a carers’ intervention on breathlessness in terms of what knowledge or information might be useful or helpful to them, and how they might learn it. Findings in relation to this final question (how they might learn) are reported in a separate paper (forthcoming).

### Data processing and analysis

Interviews were transcribed, checked and anonymised then imported into software facilitating data management, and analysed thematically using framework analysis [53]. Patients’ and carers’ transcripts were analysed separately (examining views within roles), in dyads (establishing differences/ comparability in preferences as these may impact on intervention development), and by disease group. Analysis was led by CP, with co-analysis by MF and GE to enhance validity and reliability and permit inter-analyst clarification of coding and emerging themes. This was an iterative process that began after the first two patient-carer dyad interviews were conducted.

Emergent findings were validated by the MAWG which included the operational research team, a range of clinical experts working clinically with patients with breathlessness in advanced disease and their carers (from the fields of nursing, medicine and occupational therapy across primary, community and secondary care settings), methodological experts (in qualitative methods, palliative care and breathlessness research, intervention development, psychology and informal carers) and an informal carer with experience of supporting family members with breathlessness due to cancer and COPD. This process involved presentation of the findings to the MAWG, followed by their detailed discussion. The MAWG were specifically asked to identify any surprising or unexpected findings as well as any aspects of learning about breathlessness that they felt were missing.

## Results

A purposive sample of 25 patient-carer dyads was recruited, with half the sample having a diagnosis of advanced cancer and half a diagnosis of advanced COPD; four-fifths were recruited through secondary care, and one-fifth through primary care. Table 1 reports patient-carer dyad recruitment by diagnostic group and recruiting site type and Table 2 summarises the characteristics of participating patients and carers.

**Table 1:** Patient-carer dyad recruitment by diagnostic group and recruiting site type.

Recruiting sites	Invited		Participated	
	*Cancer*	*COPD*	*Cancer*	*COPD*
**Secondary care (n = 6)**	35	25	10	10
**Primary care (n = 10)**	7	33	2	3
**TOTAL**	100		25	

**Table 2:** Characteristics of participating patients and carers.

Patients (n = 25)		Carers (n = 25)	
**Mean age:**	75 yrs (range 59–89 years)	**Mean age:**	68 years (range 42–84 years)
**Sex:**	7 female and18 male	**Sex:**	21 female and 4 male
**Diagnostic group:**	13 COPD 12 cancer (7 primary/ secondary lung cancer; 4 mesothelioma; 1 non-Hodgkin lymphoma)	**Relationship to patient:**	24 family members (20 spouses, 4 adult children) 1 friend
**Time since diagnosis (mean):**	COPD– 9 years Cancer– 2 years	**Employment:**	19 retired 5 in paid employment 1 registered disabled
**Housing:**	9 living in social housing		

Eight patients were interviewed alone, 17 with the carer present; nine carers were interviewed alone, 16 with the patient present. The interviews powerfully represented the difficult position of carers of patients with breathlessness but the absence of support for them. They identified the strong desire for an educational intervention and six interrelated topic areas for learning i.e. why they wanted to learn and what they wanted to learn about.

### Why do carers want to learn?–Need for an intervention

#### Nothing for carers

There was overwhelming evidence in the interviews of a thirst for an educational intervention on breathlessness amongst these carers: *“They do things for the patient*, *but they don’t really do anything for the carer… he’s been on courses*… *but they don’t really tell you anything”* (Carer 224; COPD). The focus of existing interventions was on patients, but carers form a dyad or unit of care with the patient in terms of day to day management, therefore focusing educational interventions on patients alone may limit effectiveness.

#### Desire to be involved

Carers wanted to be included in discussions of breathlessness management to enable them to support the patient to manage their breathlessness day to day: *“They [health care professionals] don’t necessarily ignore me but they don’t seem to talk to me… [it would be good] if a carer could be a bit more involved”* (Carer 221; COPD). They were rarely involved in advice-giving to patients or knew what the patient had been taught in terms of management strategies.

#### Recognition and affirmation

Carers sought acknowledgement of their role in supporting the patient and contributing to managing their breathlessness: *“I know the nurses and everybody and the physios and all those come and see him and do what’s right for him*, *I agree with that and we’ve had everything done that we can*. *They’ve been brilliant*. *But at the back of it*, *there’s a carer and they’re left on the back-burner”* (Carer 221; COPD).

They wanted formalised advice which strengthened their supportive role or reassured them that what they were already doing was correct: affirmation to build their confidence. They wanted information and knowledge to put them in a better position to help the patient and allay their concerns. They wanted strategies, work arounds, tips and tricks: *“You’re just sent home to deal with it on your own*, *find your own solutions”* (Carer 103; cancer).

### What do carers want to learn about?—Six interrelated topic areas

Six key topics emerged as salient for carers: 1) understanding breathlessness, 2) managing anxiety, panic and breathlessness, 3) managing infections, 4) keeping active, 5) living positively, and 6) knowing what to expect in the future. These topics were expressed either in first responses to an open question *(“What would you like to learn more about in relation to patient’s breathlessness*?*”*) or through spontaneous expression during the interviews e.g. *“what I’d really like to know is…” / “what I want to learn more about is…”*.

#### 1) Understanding breathlessness

There were two aspects that carers want to learn about in relation to understanding breathlessness: the causes of breathlessness and the experience of breathlessness. Carers wanted a better understanding of the causes of breathlessness, delivered in clear and simple language: *“I have a reasonable idea of what would happen*, *but there are… certain areas I am not very familiar with and I really haven’t had this*, *I really don’t know what emphysema is*. *I am told*, *sort of*, *the medical but sort of the layman’s terms*. *It’s never actually been explained and I’ve never asked but I would like to know”* (Carer 222; COPD). A carer of a mesothelioma patient described being shown an x-ray and not knowing what she was looking at, or what she should be looking for. For COPD patients there were opportunities for understanding COPD and its effect on the lungs, for example through attending pulmonary rehabilitation, but these carers were rarely invited to these and felt they lacked the understanding they needed.

Carers also wanted a better understanding of the experience of breathlessness—what it feels like. Some carers wanted direct experience of breathlessness whereas others just wanted a better understanding of how the patient experienced it. This desire was related in part to a need to manage their own frustration and disappointment when activities involving the patient had to be cancelled or curtailed. They felt that understanding what breathlessness feels like would help them be more empathetic and have more patience with their family member, and cope better with their own emotions around the condition. They sometimes described breathlessness as “attention-seeking” and spoke of child-like behaviour in the patient that they struggled to respond to sensitively. They felt that having a better understanding would help them respond appropriately and support their relationship with the patient: *“It would be quite helpful*… *if I could know how he felt when he was breathless… Because I would then… understand a little bit more about why… he didn’t feel he could do certain things and why he felt anxious about them”* (Carer 227; COPD).

A further source of frustration linked to limited understanding of breathlessness was the variability of the symptom on a day to day basis. Carers felt that understanding the cause of this variation might help them in turn understand how the patient was feeling: *“We could be doing the same thing every day and one day it could make him out of breath*. *I just wonder why … on certain days it makes you feel more short of breath than others… It might make me understand him better*… *how he feels”* (Carer 224; COPD).

#### 2) Managing anxiety, panic and breathlessness

There were two aspects that carers want to learn about in relation to managing anxiety and panic: how to recognise panic and how to respond confidently to panic in order to manage breathlessness. Carers described learning to recognise breathlessness that was caused, or aggravated, by panic: *“The first few times I called an ambulance [] it was simply panic… although he was gasping for breath*… *he calms down the minute I phone an ambulance… It’s visible*, *because he knows and I know help’s on its way”* (Carer 221; COPD).

Carers were uncertain about how to respond when the patient was panicking. They acknowledged that their response was often unhelpful, for example asking the patient what they could do when the patient was unable to respond due to their breathlessness: *“I don’t know what to do… when he’s bad I just keep saying*, *‘What shall I get you*, *what do you want*?*’ and he can hardly breathe like so it’s a bit naughty really isn’t it*?*”* (Carer 205; cancer). Carers themselves could feel anxious and panicked, finding it difficult and distressing to witness a breathlessness attack: *“A bit more information… because for a start it was scary*. *He would come in the bedroom in the night… ‘I can’t…’ and he couldn’t even tell me he couldn’t breathe*, *and I’m thinking what the hell*? *You just come out and you grab the phone*. *You don’t know what to expect”* (Carer 221; COPD).

They were anxious about responding helpfully; they wanted to feel confident about their response. They wanted to know what was best for the patient in that situation or reassurance they were doing the right thing. They wanted practical tools and advice—tips on how to calm the patient and their breathing: *“Good techniques for calming the person down…If you calm the system down*, *their breathing comes down*. *[…] if you can prevent the panic or reduce the panic*, *you’ve got more chance of sorting it out*, *so that would be really good… Some good tips for carers*, *especially*, *on how to calm them… a little bit of education on… what calms down the breathing”* (Carer 225; COPD).

#### 3) Managing infections

There were two aspects related to managing infections: avoiding infections and responding to infections. In the interviews this topic was predominantly identified by carers of patients with COPD; a later stage of the study (reported elsewhere), which involved workshops with patient-carer dyads living with COPD or cancer to review the emergent topics, found that avoiding and responding to infections was relevant in cancer too.

Carers wanted tips to help the patient avoid infections. Some avoided crowded places or family events and contacts involving young children, but this strategy came at the cost of compounding an already restricted and isolated life.

In terms of how to respond to infections, carers wanted to know what to do, when to start emergency medication, and when to seek additional help. Again, there were tensions within the dyads: *“do I override him*? *I can’t always treat him like a child*, *that’s not fair”* (Carer 221; COPD). Carers wanted guidance even when emergency plans were already in place.

“*I’m never too sure, you know? And I’ve always been… ‘As soon as you feel ill you don’t just take tablets’, so you’ve got to re-educate yourself really because you’re thinking, ‘Well, am I doing the right thing?’*”

(Carer 224; COPD)

“*He does annoy me. I do get angry. I’ll say to him, ‘Do you want me to call the doctor? […] because of his coughing and breathing. ‘No, I’ll leave it a few more days’ […] he’ll push it […] until I put my foot down—and then he ends up usually in [hospital]*”

(Carer 221; COPD)

The decision of whether or not to increase steroids was too much responsibility for some carers: one was concerned that it *“could have been fatal”* (Carer 225; COPD).

#### 4) Keeping active

Carers wanted practical guidance on how to help the patient to be active, including armchair exercises. They wanted to support patients in being or keeping active but this was accompanied by a difficulty in getting the balance right: knowing the appropriate amount of activity and rest. This was often a source of tension within dyads. Carers were confused about how much activity or exercise was too much or, conversely, how little was too little and should they be encouraging the patient to do more.

“*I’d like to know a little more… when should I really be stopping him from doing something […] I’ve had to say to him before ‘I’m not being unkind but if you push yourself and you really make yourself unwell’ which he has done. I am the one that looks after him and then that means I can’t get on with things because he is being silly*”

(Carer 222; COPD)

“*I’m wondering sometimes when he just sort of sits in the chair… whether that’s not helping him [], whether if he should get up and… and try and do a little bit, whether that would help him to breathe more*”

(Carer 111; cancer)

For those living with COPD there were reports of mixed messages from health care professionals about being active or not depending on the weather or on a recent exacerbation. There was also a notable mismatch in expectations between carers and patients relating to activity and this mismatch was a major cause of tension in dyads: patients felt frustrated with some feeling they wanted to do more but weren’t being allowed and that they were losing their independence, while others felt carers did not understand how they felt and had unrealistic expectations about what they could actually do. But there was also real fear on the part of carers: *“I’m always frightened that what he does is going to*… *is making his lungs wor[se] or making the cancer move more than sitting doing nothing*, *so I’d rather him sit*, *do nothing and stay as he is than do something and make him worse*, *you know*?*”* (Carer 111; cancer).

#### 5) Living positively

Carers wanted to know how to maintain a reasonable quality of life, overcoming isolation and restrictions. They wanted strategies to encourage the patient and ideas for activities that were appropriate and that they could do together—especially within couples. Many described the discovery that shopping trolleys make great walking frames as a revelation, with the bonus of not looking like a medical aid (the latter being a concern for some, as noted below). They wanted help to plan, pace and problem solve in relation to life with breathlessness. Again there was a mismatch in expectations within the dyads around what was possible and what was not.

There was also the issue of stigma. While some patients accepted help in the home or garden, or were willing to use wheelchairs or mobility scooters in order that they could do more with their carer, others were more reticent about using these leading to frustration for carers: thus this was a further source of tension within dyads. Education around this would be valued: strategies carers can use to encourage patients to have help in the home or garden, or use mobility aids, as well as information on what support or equipment was available and where to get it.

“*I feel like I'm not married, you know? Where you've always been married, all these years, and all of a sudden you’re doing it, walking about, on your own. But he’s adamant he’s not having one [mobility scooter]*”

(Carer 223; COPD)

Spousal carers talked about how they struggled to keep the patient upbeat, and how they struggled with the impact of this on their quality of life as a couple. They wanted strategies, ideas and suggestions to boost the patient’s confidence and ideas for activities they could to do together. Thus this topic was essentially was about having a life.

#### 6) Knowing what to expect in the future

Knowing what to expect in the future was about carers feeling prepared as illness progressed. It was not so much about prognostication, which is challenging in COPD in particular. Carers were very keen for information and education around possible trajectories or progression of breathlessness: not knowing was a source of worry. They wanted to be able to anticipate future changes so they could be proactive in responding rather than simply reacting as they went along. For example, some carers were discovering that they might need to make some changes to the patient’s diet as their breathlessness made eating more difficult, but they’d felt unprepared for this. Many felt unprepared for the trajectory and its downward decline, coming blind to new aspects of the conditions e.g. the potential for pneumonia or for hospital admission.

“*…to know what stages we’ve got left to expect… knowing the progression of the illness and the stages… would be very helpful*”

(Carer 225; COPD)

“*There is the worry of the future…We just don’t know where we’re going from here really. I mean what’s going to happen when you [wife/patient] deteriorate more?*”

(Carer 226; COPD)

“*…understanding what to expect […] how they’re going to be or [that] there are going to be good days, bad days, flare ups; you don’t know these things until they actually happen*”

(Carer 224; COPD)

Some carers also noted that patients too lacked this knowledge: *“…if you know at the beginning of the stages and what would happen and this sort of thing then I think you would cope better because you've already been forewarned… There's no good going through with blinkers ‘cause that's not going to help you*. *And if you know what to expect when it happens then you can both cope better*. *But neither of you know*. *And it does come a bit hard”* (Carer 223; COPD).

However for most carers there was a tension relating to their desire to know more about what to expect in the future, but not wanting to upset the patient. In some cases they did not want the patient to learn this with them as they felt it would cause further anxiety for patient.

For those caring for cancer patients (with primary lung or metastatic lung cancer) there was also uncertainty around breathlessness symptoms and the extent to which any deterioration could or should be associated with disease progression. Even when the underlying cause of breathlessness had been explained as not being associated with tumour growth or spread it was hard for carers and patients to separate breathlessness symptoms from cancer symptoms. This in turn led to heightened anxiety for both carer and patient.

### Disease group differences

Although both groups wanted to learn about all of the six topics outlined above, there were some differences by disease group. Cancer carers regarded increasing breathlessness as a sign of disease progression and were particularly interested in learning how to manage breathlessness episodes: an aspect of caring that was relatively new to them and a response to a patient need.

“*If you could be sort of told if there’s anything you can do for them. At the moment I wouldn’t know… I mean if we got a panic attack or anything I wouldn’t know what to do, I’m just sort of standing here looking at him because I wouldn’t know whether there was anything I could do*”

(Carer 211; cancer).

COPD carers were particularly interested in learning how to manage infections and in managing their own anxieties related to caring: these are aspects of caring related to both patient needs and carer needs. For example the responsibility of knowing when to start emergency medications was a concern that was frequently mentioned: *“we never quite know […]… it’s like a balancing act whether to start the steroids or … I know they say leave it 24 hours*, *but you’re still a bit unsure… you know*?*”* (Carer 224; COPD).

Thus for carers of patients with cancer the driver for the need for knowledge or education appeared grounded in their desire to help the patient, whereas for carers of patients with COPD the driver was meeting the needs both of the patient and of the carer.

## Discussion

This paper reports the need and desire for an educational intervention for carers of patients with breathlessness in advanced disease: this appetite resonates with existing literature relating to other carers [54]. In addition to identifying their need for advice and reassurance this paper further identifies the lack of support for these carers despite their wish to be acknowledged and involved. Importantly it identifies and describes six key topics carers of patients with breathlessness in advanced disease want to learn about in order to help them support their family member or friend: 1) understanding breathlessness, 2) managing anxiety, panic and breathlessness, 3) managing infections, 4) keeping active, 5) living positively, and 6) knowing what to expect in the future.

Carer views are paramount to the development of an educational intervention for carers, but patient views also play a role. Patient views were largely similar to carer views, but areas of divergence or tension within the patient-carer dyads were identified. A theme common to most of the six topics was mismatched expectations and the management of resulting tensions within dyads: a long-established characteristic of the caring role [55]. There was a strong desire to be enabled to do things together, as a dyad. The six topics, and this cross-cutting theme of relationship-management, have resonance with the existing literature on carer experiences and needs in advanced disease (e.g. [56,57]) and more specifically in advanced COPD (e.g. [13,28,58,59]), cancer (e.g. [60,61]) and breathlessness (e.g. [39]). Further, although primarily geared to patients, the suggested educational components of pulmonary rehabilitation programmes for adults [62] can be mapped onto these six topics identified for carers.

There was a sense from these carers that the patient-carer dyad was not regarded by health care professionals as the unit of care. This was surprising given the central role carers play in caring for breathless patients, but echoes existing literature noting the need for greater acknowledgement and recognition of carer expertise [63–65]. The patient and carer interviews rarely referred to carers as part of health care professionals’ response to breathlessness: carers were rarely involved as potential experts in patient care, they rarely understood breathlessness as they had not been taught about it, they rarely knew how to get right the balance of activity and rest, and they rarely described being included in strategies to live positively. They were on the periphery. Carers suggested that knowledge-gains on the topics would enable them to better support the patient and manage their breathlessness, but would also support themselves in terms of managing their own frustrations, anxieties, and quality of life as a carer.

There were some differences in learning needs by disease group, with a greater interest among cancer carers in gaining knowledge for their role in supporting the patient whereas for COPD carers their desire related equally to their own needs as carers: these differing of aspects of the caring role have been referred to as direct care needs and enabling needs [66] and reflect the dual role carers have as both co-workers and clients [67]. These diagnostic group differences in learning needs can probably be explained by the shorter duration of the caring role in cancer such that cancer carers appeared less ground down by the caring experience, and the impact of restrictions and isolation, than those caring for patients with advanced COPD. Thus the needs of cancer carers related predominantly to the needs of the patient.

A limitation of this study is that the majority of our dyads were recruited through secondary care; recruiting dyads through primary care proved more challenging. It may be that the recruitment approach varied between the two settings, with more personal hand over of recruitment packs by secondary care clinicians, or patients who only accessed primary care may have been less likely to identify family, friends or carers as needing an intervention on breathlessness. Dyad recruitment was conducted via the patient and we were mindful that patients accessing secondary care may differ from those with only primary care access. However it could be argued that there is a benefit of recruiting predominantly from secondary care given that patients recruited through this source are likely to be at higher risk, with more health care utilization and more severe exacerbations: the educational needs of their carers may therefore be greater. Regardless of whether this sampling strategy was a strength or a weakness, the remarkable similarity in the characteristics of our sample of carers (age, sex and relationship to the patient) to samples of carers of patients with breathlessness recruited to other studies [12–14] and the resonance of the findings with the existing literature noted above provides some reassurance.

Recruiting carers via patients is potentially a further limitation. The study recruitment materials stated clearly that carers could participate without the patient, however this still relied on patients passing on the recruitment pack to carers. Patients could therefore act as gatekeepers to the participation of carers [68]. However it is again reassuring that, despite this approach, tensions within the patient-carer dyads were still identified. Further, later stages of the study recruited carers directly from support groups to participate in workshops to co-develop the intervention (paper forthcoming). At these workshops carers were asked to review the findings of the carer and patient interviews presented here and were asked to identify any surprising or unexpected findings as well as any aspects of learning about breathlessness that they felt were missing: no new areas were identified.

The findings were broadly consistent across interview type: in both patient-carer dyad interviews and separate patient and carer interviews. A future paper will outline how these data, and data collected contemporaneously relating to carers’ preferences for intervention delivery mode, has informed early development of an educational intervention for carers of patients with breathlessness in advanced disease.

## Conclusions

This study is the first to describe in detail the unmet educational needs of carers of patients with breathlessness in advanced disease. It identified the need and desire for an educational intervention for carers, and six key topics carers want to learn about in relation to their caring role. Meeting the educational needs of carers requires robustly developed and evaluated interventions; this study provides the evidence-base for the content of an educational intervention for carers of patients with breathlessness in advanced disease.
